# Utility of T2* and DWI-IVIM to distinguish inflammatory and noninflammatory strictures in Crohn’s disease: a prospective cross-sectional cohort study

**DOI:** 10.1186/s41747-026-00715-0

**Published:** 2026-04-22

**Authors:** Kim Johanna Beek, Kyra van Rijn, Floris de Voogd, Nienke Petronella Maria Wassenaar, Christianne Buskens, Jarmila van der Bilt, Willem Bemelman, Geert Renaat D’Haens, Krisztina Barbara Gecse, Karin Horsthuis, Jeroen Tielbeek, Aart Mookhoek, Andra Neefjes-Borst, Oliver Gurney-Champion, Jaap Stoker

**Affiliations:** 1 Department of Radiology and Nuclear Medicine, Amsterdam UMC, location University of Amsterdam, Amsterdam, The Netherlands; 2 Amsterdam Gastroenterology Endocrinology Metabolism, Amsterdam, The Netherlands; 3 Department of Gastroenterology and Hepatology, Amsterdam UMC, location University of Amsterdam, Amsterdam, The Netherlands; 4 Department of Surgery, Amsterdam UMC, location University of Amsterdam, Amsterdam, The Netherlands; 5 Department of Radiology, Spaarne Gasthuis, Haarlem, The Netherlands; 6 Institute of Tissue Medicine and Pathology, University Bern, Bern, Switzerland; 7 Department of Pathology, Amsterdam UMC, location University of Amsterdam, Amsterdam, The Netherlands

**Keywords:** Biomarkers, Crohn’s disease, Diffusion magnetic resonance imaging, Magnetic resonance imaging, Constriction (pathologic)

## Abstract

**Objective:**

In Crohn’s disease (CD), noninflammatory strictures benefit from surgical resection or endoscopic dilatation, whereas inflammatory strictures might still respond to anti-inflammatory treatment. Therefore, it is crucial to determine whether a stricture is inflammatory or noninflammatory. We investigated whether magnetic resonance imaging (MRI) T2* and intravoxel incoherent motion (IVIM) parameters can distinguish inflammatory from noninflammatory strictures, using histopathology of location-matched surgical resection specimens as reference.

**Materials and methods:**

CD patients scheduled for small bowel segment resection were included. MRI scans were acquired in 32 patients, including conventional anatomical T2*-weighted and DWI-IVIM sequences. The field of view was centred on the most significant small bowel stricture. T2* and IVIM parameters were compared with location-matched surgical resection specimens on histopathological sections, scored as inflammatory or noninflammatory. A Mann–Whitney *U* test (α = 0.01) was used to assess differences in MRI parameters between inflammatory and noninflammatory sections.

**Results:**

Thirty-one patients (55% females) were analysed. Median T2*-value was significantly higher in inflammatory sections (25.4 ms [interquartile range 19.0‒33.1], *n* = 33) *versus* noninflammatory sections (18.6 ms [14.5‒27.8], *n* = 26) (*p* = 0.010). Diffusion coefficient was lower in inflammatory sections (0.0012 mm²/s [0.0010‒0.0014], *n* = 35) *versus* noninflammatory sections (0.0014 mm²/s [0.0011‒0.0019], *n* = 24) (*p* = 0.039). No significant differences were found in pseudodiffusion coefficient and pseudodiffusion signal fraction (0.06 mm²/s [0.03‒0.08] *versus* 0.06 mm²/s [0.03‒0.11], *p* = 0.926; 0.15 [0.09‒0.20] *versus* 0.18 [0.11‒0.24], *p* = 0.327).

**Conclusion:**

A significantly higher T2*-value and a trend towards a lower diffusion coefficient were observed in inflammatory sections compared to noninflammatory sections in CD. External validation is needed.

**Relevance statement:**

Higher T2*-value and lower IVIM diffusion coefficient could potentially serve as a biomarker in inflammatory stricture sections compared to noninflammatory sections in Crohn’s disease.

**Trail registration:**

Dutch trial register NL9105: https://trialsearch.who.int/Trial2.aspx?TrialID=NL-OMON54948.

**Key Points:**

- T2* could serve as a biomarker for distinguishing inflammatory from noninflammatory strictures in Crohn’s disease.
- Diffusion measured by IVIM can distinguish inflammatory from noninflammatory strictures in Crohn’s disease.
- Muscularisation was more prominent than fibrosis in strictures due to Crohn’s disease.

**Graphical Abstract:**

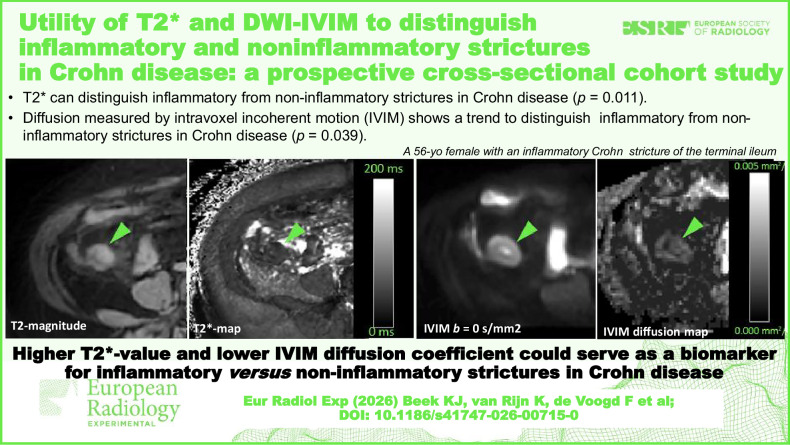

## Background

Crohn’s disease (CD) is a chronic autoimmune disorder, characterised by episodes of relapse alternating with periods of remission [1]. Inflammation leads to the activation of the tissue repair mechanism, which results in an increase in the extracellular matrix in the bowel wall. Although this process is activated in physiological wound healing, it becomes uncontrolled in CD with fibrosis, muscular hypertrophy and hyperplasia and an increase of fat and nerve content in the bowel wall [2]. This causes bowel wall thickening and luminal narrowing, which can result in a stricture [3]. Half of patients with CD develop a stricture as a complication during their disease course [4].

Strictures are traditionally classified as either inflammatory or fibrotic. However, the focus on fibrosis as the hallmark of chronicity is a simplification of stricture composition. Recent literature shows that muscular hypertrophy/hyperplasia plays a crucial role in stricture formation [5, 6] that presumably oversteps that of intestinal fibrosis. By considering the relative contribution of inflammation, fibrosis and muscular hypertrophy/hyperplasia in strictures, a better distinction might be possible for strictures refractory (*i.e*., classified on histopathology as noninflammatory) *versus* not refractory for anti-inflammatory treatment (*i.e*., classified on histopathology as inflammatory). This distinction could also be helpful for the development of new treatment targets for strictures refractory to anti-inflammatory treatment.

Several studies have investigated conventional MRI parameters (used in the clinical setting for assessing CD) to distinguish inflammatory and fibrotic strictures [7–13]. These studies showed an overlap between parameters that correlate with inflammation and fibrosis. However, conventional MRI does not characterise underlying tissue microstructures, which may be relevant when classifying strictures. Therefore, over the last decade, there has been a growing interest in investigating quantitative MRI parameters to quantify fibrosis. Such parameters directly probe and quantify tissue microstructure properties. Promising results have been published in single-centre studies concerning magnetisation transfer imaging [14], delayed contrast enhancement [12], T2* [15] and intravoxel incoherent motion (IVIM) [16]. Magnetic transfer imaging showed fewer assuring results in a multicentre study [17]. The T2* relaxation time, representing the T2* decay over time in ms, and the perfusion fraction, measured by intravoxel incoherent motion (IVIM), representing the signal fraction from capillaries, showed promising results for the detection and grading of fibrosis in single-centre studies, with the additional benefit of not using an intravenous contrast agent [15, 16].

Only focusing on fibrosis would be an oversimplification of stricture composition. Therefore, we investigate whether T2* and IVIM can be used to distinguish inflammatory (*i.e*., inflammatory and mixed) from noninflammatory strictures, using surgical histopathology as a reference standard.

## Methods

### Study design

A prospective, observational cross-sectional cohort study was performed in CD patients undergoing surgical small bowel resection (Dutch trial register NL9105: https://trialsearch.who.int/Trial2.aspx?TrialID=NL-OMON54948). Patients were recruited from December 2019 until March 2022, and all provided written informed consent. The study was conducted in agreement with the Declaration of Helsinki and approved by the institutional Ethics Review Board of the Amsterdam University Medical Centre, location Academic Medical Centre.

### Patients

Eligible adult CD patients (≥ 18 years) scheduled for surgical small bowel resection were included. The indication for small bowel resection was determined in a multidisciplinary team meeting. Exclusion criteria were the presence of an isolated colonic stricture, pregnancy, inability to give informed consent, ongoing gastroenteritis, or contraindications for MRI. Patients were treated according to local treatment protocol, and this study did not have an influence on the patient’s treatments, since it concerns an observational cross-sectional study.

### MRI protocol

All patients underwent magnetic resonance enterography within 12 weeks prior to surgical small bowel resection. Due to restrictions, this interval was prolonged in three patients during the COVID pandemic (14 weeks, 16 weeks and 25 weeks).

Patients prepared for the scan in accordance with the standard clinical magnetic resonance enterography procedure, fasting for 4 h followed by drinking up to 1,600 mL of 1.8%-mannitol-solution (Baxter BV) as an intraluminal contrast agent in the 60 min before the magnetic resonance enterography. Images were acquired in supine position using a 3-T MRI (Ingenia, Philips, Best, the Netherlands) with a 16-channel anterior and 16-channel posterior coil. The imaging protocol is summarised in Table 1.

**Table 1:** Imaging protocol parameters

Sequence	FOV (mm*mm)	Orientation	FS	Acquired/reconstructed voxel sizes (mm*mm)	Parallel imaging factor/ partial fourier factor	2D multislice/3D	Flip angle (°)	*N* slices/slice thickness (mm)/slice gap(mm)	TR/TE (ms)	B-values and the number of directions/averages in brackets (s/mm^2^)	Acquisition time
T2*: Multi-echo spoiled GE	400*400	Axial	SPAIR	2*2/1.4*1.4	1 (AP); 2 (FH)/1	3D	25	65/2.3/NA	51.8/Sixteen different TE: 2.0, 5.1, 8.3, 11.4, 14.6, 17.7, 20.8, 24.0, 27.1, 30.3, 33.4, 36.5, 39.7, 42.8, 46.0, 49.1		5 min 36.8 s
IVIM: Diffusion-weightedSS-EPI	450*118.75	Axial	SPAIRGradient reversal	2*2/1.6*1.6	1.4/0.7	2D	90	24/5/1	5,000/56	18 b-values:0 (9x), 1 (3x), 2 (3x), 5 (3x), 10 (3x), 20 (3x), 30 (3x), 40 (3x), 50 (3x), 75 (3x), 100 (3x), 150 (3x), 200 (3x), 300 (3x), 400 (3x), 500 (3x), 600 (3x), 700 (3x)	5 min 5 s
Balanced GE	450*450	Coronal	No	1.5*1.8/0.9*0.9	2/1	2D	60	26/5/1	2.5/1.3		29.7 s
T2-weighted Ultra fast SE	400*400	Axial	No	1.6*2.0/0.8*0.8	2.5/0.6	2D	90	96/4/5	759/118.9		1 min 12.9 s
T2-weighted TSE	400*300	Axial	SPAIR	1.3*1.6/1.1*1.1	2.5/1	2D	90	68/7/1	1,072.8/50		1 min 11.6 s
T1-weighted spoiled GE before and after Gd	450*405	Coronal	SPAIR	2*2/1.9*1.9	1.8 (RL); 2 (AP)/0.8 (RL); 0.9 (AP)	3D	10	90/2/NA	2.4/1.06		17.1 s
T1-weighted spoiled GE after Gd	380*380	Axial	SPAIR	1.6*1.6/1*1	1.5/1	3D	10	272/2/NA	2.5/1.2		57.2 s

*FOV* Field of view, *FS* Fat saturation, *GE* Gradient echo, *N* Number, *NA* Not applicable, *SE* Spin-echo, *SPAIR* Spectral attenuated inversion recovery, *SS-EPI* Single-shot echo-planar imaging, *TE* Echo time, *TR* Repetition time, *TSE* Turbo spin-echo

Quantitative MRI scans were obtained during free breathing without motion compensation. IVIM was obtained using a multislice diffusion-weighted single-shot echo-planar imaging sequence. Prior to the T1-weighted postcontrast sequence (part of the local standard clinical magnetic resonance enterography protocol), 20 mg of antiperistaltic medication (scopolamine butylbromide, Buscopan; AS KALCEKS) and 0.1 mmol/kg of contrast agent (Dotarem, Gadoteric acid, Guerbet) were intravenously administered.

### Image analysis

The T2*-value was derived from the T2*-weighted sequence and represents the T2* decay over time (ms). T2* represents the relaxation of the transverse component of the signal. The faster the T2* decay, the lower the T2*-value. Multiple components can contribute to T2*-values, including local field heterogeneity, but in the context of CD, it is considered a marker for fibrosis [15].

Diffusion coefficient (*D*), pseudodiffusion coefficient (D*) and pseudodiffusion signal fraction (f) were derived from the IVIM sequence. *D* represents the amount of diffusion in a voxel. D* reflects dephasing due to incoherent motion, which is typically attributed to perfusion in semirandomly organised capillaries for easy clinical interpretation. In this case, *f* (typically called perfusion fraction) represents the signal fraction from capillaries.

### Matching between MRI and histopathology

**Fig. 1:**
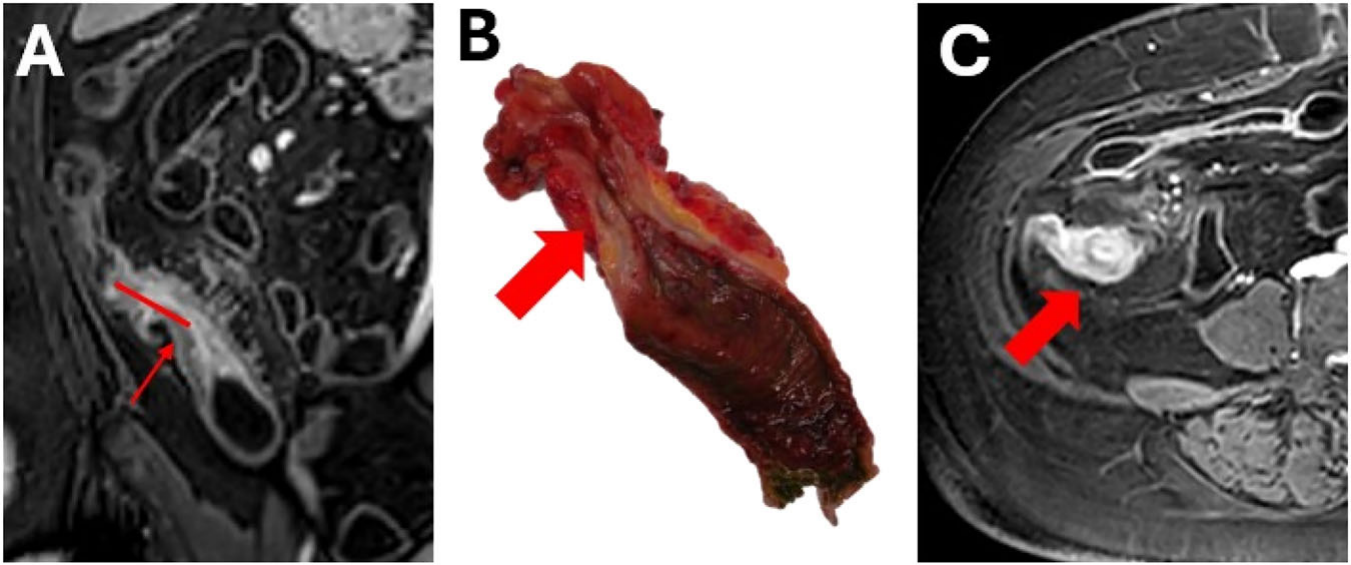
Matching between magnetic resonance imaging and histopathology. **A** Coronal T1-weighted postcontrast image showing the distance (red line, 3 cm) between the ileocecal valve and the most narrowed part of the stricture. The red arrow indicates the most narrowed part of the stricture, where all measurements were performed. **B** Corresponding fresh histopathological resection specimen. **C** Axial T1-weighted postcontrast image at the level where the measurements were performed

The narrowest locations per stricture (one to four, based on length) were identified on a T1-weighted postcontrast image by two clinical researchers (K.R.: 6 years of experience in MRI of CD, K.B.: 3 years of experience in MRI of CD). The distance from the narrowest section to the ileocecal valve or ileocolonic anastomosis was recorded. The selection of the sections and distances was supervised by an abdominal radiologist (J.S.) with 30 years of MRI experience in CD. Locations were marked to match histopathological specimens, using the ileocecal valve/anastomosis as reference. If multiple strictures were present, at least 5 cm of unaffected bowel between them was required to define a separate stricture. The matching between MRI and histopathology is visualised in Fig. 1.

### Analysis of quantitative MRI data

**Fig. 2:**
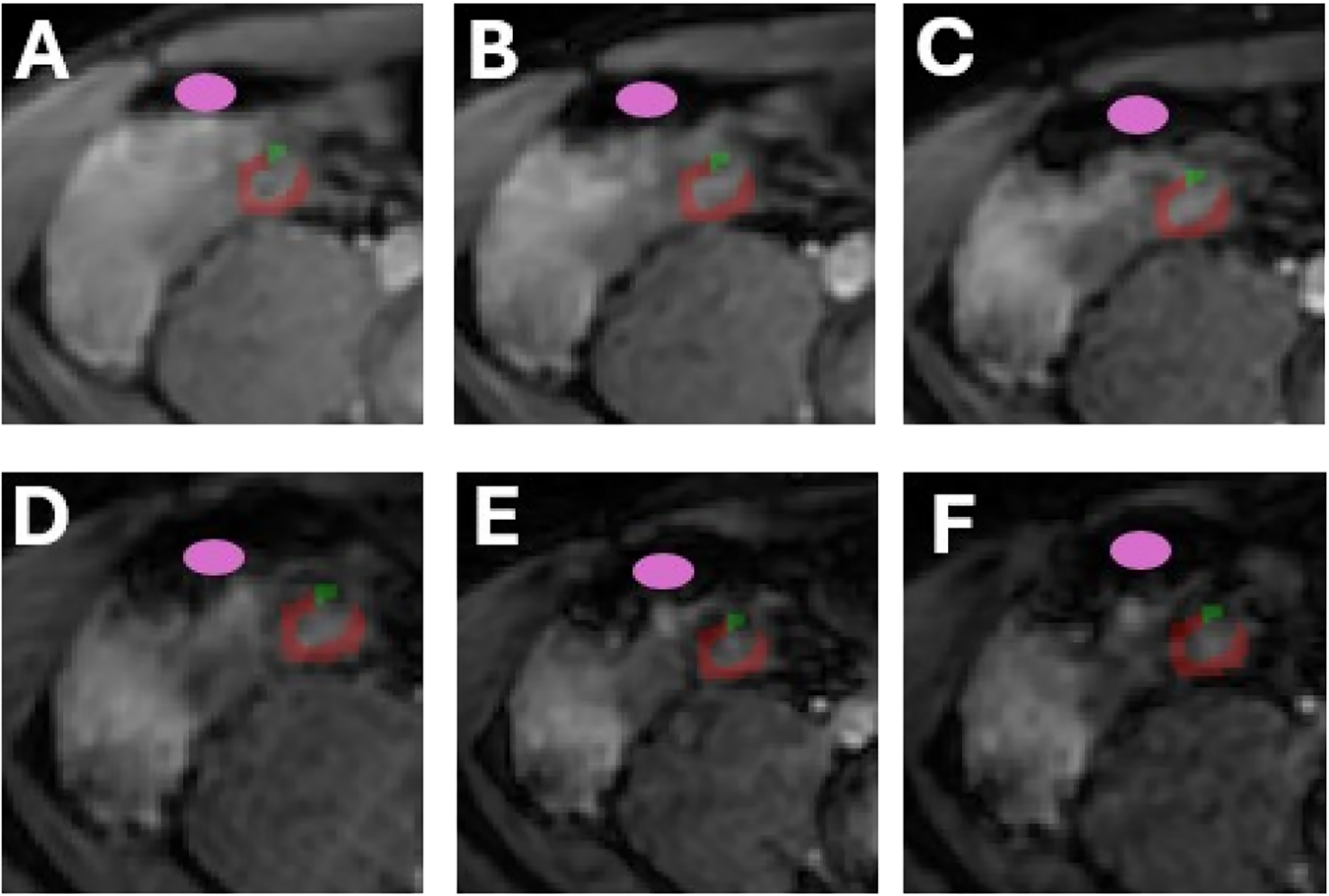
Example of region-of-interest placement on anatomical T2* images. The ileocecal junction is imaged on the anatomical T2* images (**A**‒**F**) with the red segmentation indicating the most narrowed part of the stricture located at the terminal ileum, and the gas-tissue transition zone segmented in green. The green segmentation was excluded from the analysis. The first echo time is labelled on **A**, extending through to the sixth echo time on **F**. The pink point represents the gas in the caecum, which is enlarging over time

Segmentations were performed manually by a clinical researcher (K.B.) in ITK-SNAP (version 4.3.0, 30 November 2015). Regions-of-interest (ROIs) were placed at the narrowest locations of the stricture. The ROI encompassed the entire bowel wall on a single slice of the T2* (echo time = 2.0 ms) and IVIM (*b* = 0 s/mm^2^) magnitude data. ROIs were checked for anatomical correctness by a second clinical researcher (K.R.). ROIs were manually adjusted per echo time or *b*-value for movement to exclude regions into which other tissue moved. For T2* segmentation, gas-tissue transition zones were separately segmented and subtracted from the bowel wall segmentations to prevent gas-tissue artefacts influencing T2*. This is visualised in Fig. 2. T2* is very sensitive to magnetic field heterogeneities, and such heterogeneities can mask the effect of fibrosis. Therefore, we aimed to exclude heterogeneities arising from metal artefact (*e.g*., due to surgical clips) or gas-tissue transition zones due to bowel movements. Such regions were discussed by a clinical researcher (K.B.) with an MRI physicist (O.G.C.), and if needed, studied on later echoes. If the gas-tissue transition artefact or metal artefact obscured the ROI and hindered adequate measurement, the section was excluded.

Only the first six echo times were used for analysis, as a substantial amount of signal was gone after six echoes, and as later echoes showed unexpected signal enhancements in some voxels, resulting in poor fits and unexpectedly high T2*-values. T2* parameter maps were obtained with Matlab (version 2021a, The MathWorks) by means of in-house software that performs voxel-wise fitting using a monoexponential fit [18]. For IVIM, deformable image registration (motion correction) was performed in Elastix using principal component analysis-based groupwise image registration [19–21]. *D*, D* and *f* maps were generated using voxel-wise fitting with the biexponential segmented fit from https://github.com/oliverchampion/IVIMNET/fitting_algorithms/fit_segmented [22], implemented in Python (version 3.6). Voxels with f < 0.001 were excluded from the pseudodiffusion coefficient ROI characteristics [23], as the pseudodiffusion coefficient is undefined for nonperfused voxels. To exclude outliers (*e.g*., due to partial voluming, noise and remaining motion), the per-section median parameter values from within the ROI were reported.

### Collection of conventional MRI data

This section is included in the Supplementary Materials and Methods section and in Table S1.

### Histopathological analysis

A clinical researcher (K.B., K.R. or F.V.) was present when the resected small bowel specimen arrived at the pathology department. The specimens were opened lengthwise, and the narrowest stricture areas were marked with pins in concordance with the MRI. After photographing the specimens, these underwent formalin fixation for 24 h. Tissue blocks were taken from the marked sites, and serial sections were stained with haematoxylin and eosin, smooth muscle actin and trichrome. Two experienced gastrointestinal pathologists, with 8 (A.M.) and 33 (A.N.) years of experience, scored the sections independently. The Nancy index [24] was used to assess mucosal inflammation. A separate score was used to measure submucosal fibrosis (0 = none, 1 = mild, 2 = moderate, 3 = marked). Thickening of the muscularis mucosae and muscularis propria was assessed with the smooth muscle actin stain (0 = none, 1 = mild, 2 = moderate and 3 = marked). The extent of adipose tissue in the submucosa was assessed on haematoxylin and eosin as either 0 (none or mild) or 1 (moderate or marked).

In parallel to scoring these items, the predominant phenotype of the stricture was determined. This determination was performed by assessing the entire bowel thickness in a single section and based on which item or items contributed to the increase in wall thickness the most. Three stricture phenotypes were identified: (1) predominantly inflammatory with extensive inflammation in the different layers of the bowel; (2) predominantly chronic with submucosal fibrosis, submucosal adipose tissue, thickening of the muscularis mucosae, thickening of the muscularis propria or a combination of these items; or (3) a mixed phenotype where both inflammation and chronic changes contributed to the increase in bowel wall thickness more or less equally. As the entire thickness of the bowel wall was assessed, a severe active inflammation of the mucosa as assessed by the Nancy index, did not automatically translate to predominantly active mixed phenotype.

In cases of disagreement between pathologists, a consensus meeting determined the final score. If multiple sections were required to assess the entire bowel wall circumference, the highest score (*i.e*., fibrosis, muscularisation, Nancy) of those sections was used for analysis. If different phenotypes were identified, the bowel wall was classified as mixed. The consensus score was used for subsequent data analyses. Predominantly inflammatory and mixed sections were grouped as inflammatory and compared with predominantly chronic (noninflammatory) sections.

### Statistical analysis

Statistical analysis was conducted using SPSS version 28 (IBM Corp.). Continuous data were reported as mean ± standard deviation or as median [interquartile range] for skewed data, while categorical data were presented as numbers (%).

MRI variables were compared between noninflammatory and inflammatory (inflammatory and mixed) sections. Cutoff values were determined for significant results with receiver operating characteristic (ROC) curves based on the Youden index. Continuous variables were analysed using either a *t*-test or Mann–Whitney *U* test based on normality or non-normality. Binary variables were assessed with a χ^2^ test, and ordinal and nominal variables with a χ^2^ test for trend. Interobserver agreement for MRI and histopathological scoring was assessed with Cohen’s weighted κ for ordinal variables with quadratic weighing and intraclass correlation coefficients with a two-way random model for absolute agreement for continuous variables. Due to the exploratory nature of the study, no *post hoc* correction was performed. The significance level for our main question was adjusted to a *p*-value of < 0.01 using a Bonferroni correction, due to performing four statistical tests. For the other tests, a *p*-value of < 0.05 was considered statistically significant. No correction for clustering of tissue sections within patients was performed due to the heterogeneity of tissue sections.

## Results

### Population

A total of 32 consecutive patients (Table 2) with 37 strictures (five patients with two strictures) were included. Of these 37 strictures, 74 sections were scored on conventional MRI and on histopathology for the predominant histopathological feature. One patient with one stricture (two sections, resection specimen consisted of colon tissue only) was excluded, and another section was scored as normal bowel by histopathology (*n* = 1), leaving 31 patients and 71 sections in the analysis. For T2* and IVIM, 12 of the 71 sections were excluded and 59 sections were analysed. For T2*, exclusions came from: (1) gas artefacts causing very short T2* times (*n* = 7); (2) affected bowel wall could not be identified on magnitude T2* data (*n* = 3); (3) Presence of metal artefact which influenced T2* measurement (*n* = 1); and (4) affected bowel wall was not included in field of view at T2* acquisition (*n* = 1). For IVIM, exclusions came from: (1) too much bowel wall motion between different *b*-values remained after image registration to ensure reliable segmentation (*n* = 7); (2) the affected bowel wall was not identified on magnitude IVIM data (*n* = 4); and (3) affected bowel wall was not present in the field of view at IVIM acquisition (*n* = 1).

**Table 2:** Baseline characteristics

Characteristics in median [IQR], mean (± SD) or *n* (%)	Patients with stricturing CD (*n* = 31)
Sex (*n*, % of female)	17 (55%)
Age (years)	36 [25–59]
Smoking status	
Never smoked	17 (55%)
Previous smoker	8 (26%)
Current smoker	6 (19%)
BMI in kg/m^2^	24 [21–27]
Harvey Bradshaw Index	7 [3–9]
Crohn’s disease obstructive score	4 [0–5]
Nausea and vomiting	14 (45%)
Dietary restrictions	15 (48%)
Hospitalisation due to obstruction	1 (3%)
Disease duration (years)	7 [2–12]
Anti-inflammatory medication	
None	10 (32%)
Corticosteroids monotherapy or add-on	1 (3%)/5 (16%)
Thiopurines	5 (16%)
Add-on methotrexate	2 (6%)
Biologicals	15 (48%)
Indications for surgery	
Obstructive symptoms	12 (39%)
Therapy refractory	10 (32%)
Therapy refractory and obstructive symptoms	3 (10%)
Early counselling for ileocecal resection	3 (10%)
Surgery is treatment of preference*	1 (3%)
Therapy refractory and penetrating disease	1 (3%)
Obstructive symptoms, therapy refractory and penetrating disease	1 (3%)
Type of surgery	
Ileocecal resection	20 (65%)
Ileocecal re-resection	9 (29%)
Ileocecal resection and small bowel segment resection	1 (3%)
Ileocecal resection and stricturoplasty	1 (3%)
Time between MRI and surgery (days)	21 [4–56]
Surgical history	9 (29%)
Ileocecal resection	9 (29%)
Ileocecal re-resection	1 (3%)
Stricturoplasty	1 (3%)
Subtotal colectomy	1 (3%)
History of endoscopic dilatation	4 (13%)
Age at diagnosis (years)	
A1, ≤ 16	2 (6%)
A2, 17–40	20 (65%)
A3, > 40	9 (29%)
Disease location	
L1, ileal	15 (48%)
L2, colonic	0
L3, ileocolonic	16 (52%)
+ Perianal disease	6 (19%)
Disease behaviour	
B1, non-stricturing/non-penetrating	2 (6%)
B2, stricturing	25 (81%)
B3, penetrating	4 (13%)
Faecal calprotectin (mg/g) (*n* = 18)	839 [258–1,853]
CRP (mg/L) (*n* = 25)	7 [3–13]
Patients who underwent endoscopy within the last 6 months	17 (55%)
Stricture on endoscopy	13 (42%)
Possibility to intubate stricture	3 (10%)

*BMI* Body mass index, *CRP* C-reactive protein

* The patient had a history of leukaemia

### Histopathology

Thirty-eight sections (58%) were scored as inflammatory (eight sections (11%) as inflammatory and 30 (47%) as mixed) and 33 (42%) as noninflammatory. Median Nancy index (*n* = 71) was 4.0 [3.0‒4.0], the median fibrosis score (*n* = 71) was 1.0 [1.0‒2.0], the median score of muscularisation of the muscularis mucosae (*n* = 71) was 2.0 [1.0‒2.0], and of the muscularis propria (*n* = 70) was 1.0 [0.0‒1.0]. One smooth muscle actin stain could not be assessed at the level of the muscularis propria due to the presence of a fistula and a tangential cut. Forty-six (65%) sections had moderate or marked adipocytes in the submucosa, while twenty-five (35%) had none or mild adipocytes.

**Fig. 3:**
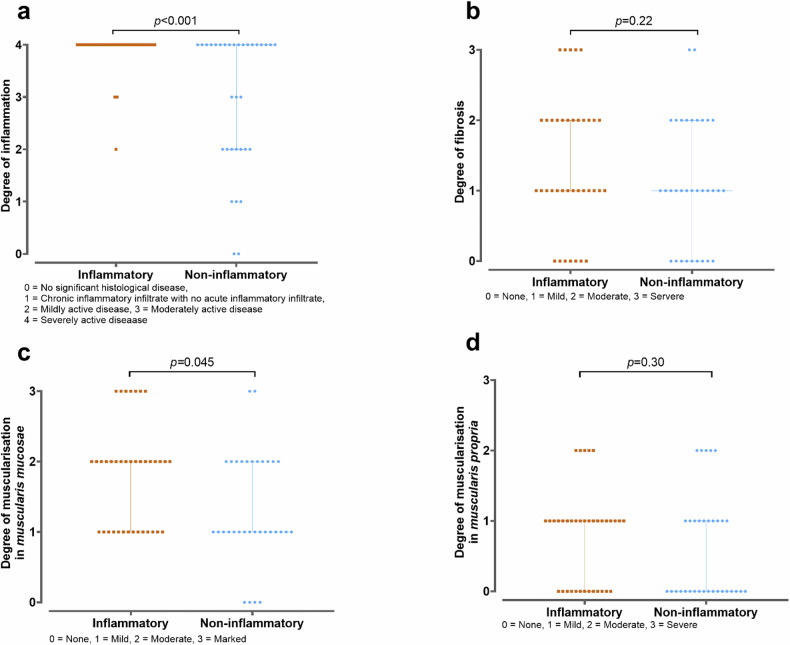
Histology: distribution of inflammatory (brown) sections and noninflammatory (blue): (**a**) degree of inflammation (Nancy index); (**b**) degree of fibrosis; (**c**) muscularisation of the muscularis mucosae; and (**d**) muscularisation of the muscularis propria

The median Nancy index was significantly higher in the inflammatory compared to the noninflammatory section (4.0 [4.0‒4.0] *versus* 4.0 [2.0‒4.0]). No difference was found in the amount of adipocytes between inflammatory and noninflammatory sections (*versus* 24, 52% *versus* 22, 48% for moderate or marked adipocytes, *p* = 0.807). Individual scores (*i.e*., fibrosis and muscularisation scores) per section, categorised in inflammatory *versus* noninflammatory sections, are visualised in Fig. S1. The median scores for inflammation, fibrosis and muscularisation across histopathological phenotypes are displayed in Fig. 3, with inflammatory sections showing higher muscularisation of the muscularis mucosae than noninflammatory sections (Fig. 3c).

### Comparison of T2* parameters with histopathological subtypes

**Fig. 4:**
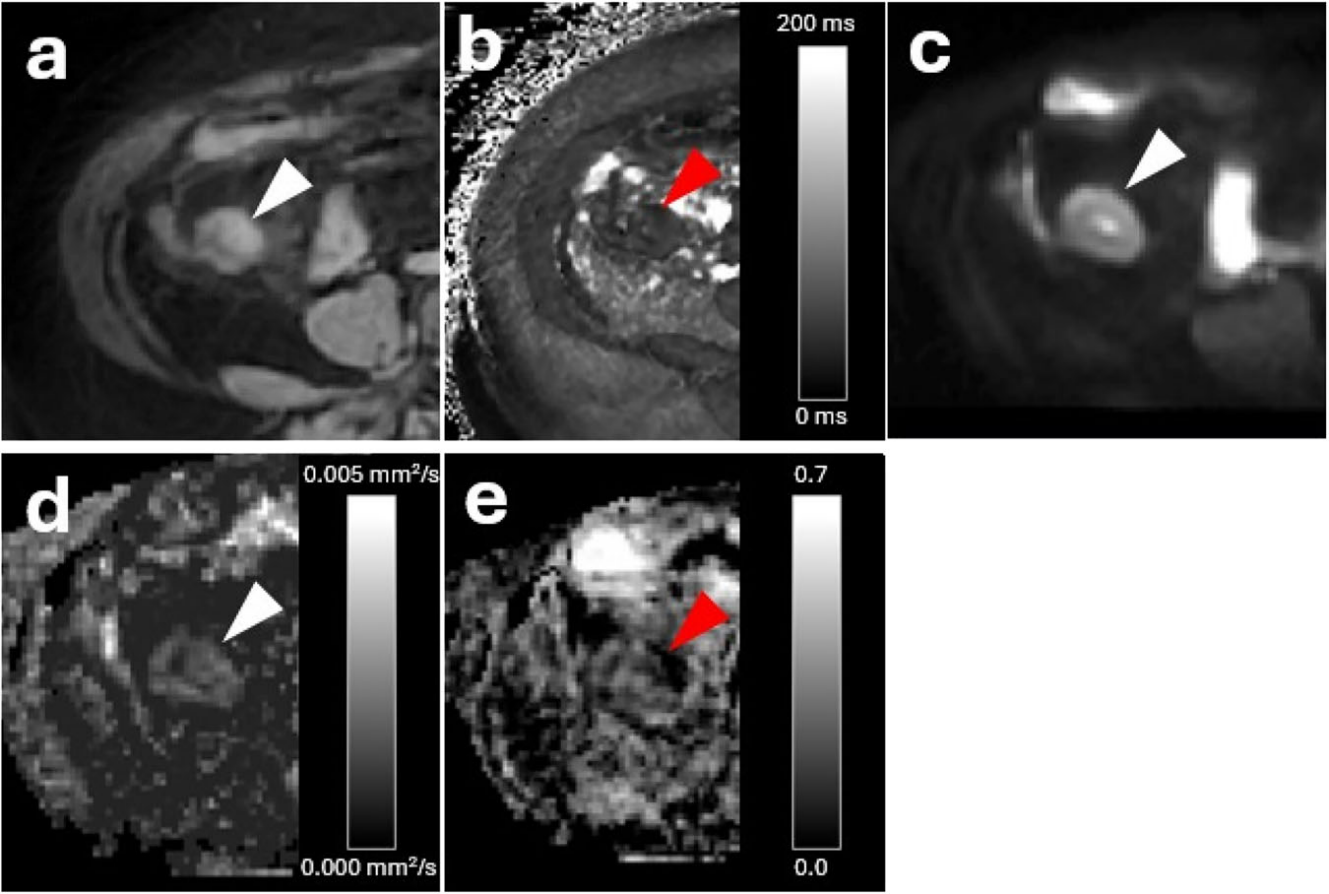
Examples corresponding with a 56-year-old female with an inflammatory stricture at terminal ileum: **a** arrowhead indicating the bowel wall on T2*-magnitude image (echo time 2.0 ms); **b** arrowhead indicating the bowel wall on T2*-parameter map with a median T2*-value of 19.6 ms; **c** arrowhead indicating the bowel wall on intravoxel incoherent motion magnitude image (b = 0 mm^2^/s); **d** arrowhead indicating the bowel wall on the diffusion parameter map with a median diffusion coefficient of 0.0007 mm²/s; **e** red arrowhead indicating the bowel wall on the perfusion fraction parameter map with a perfusion fraction of 0.20

**Fig. 5:**
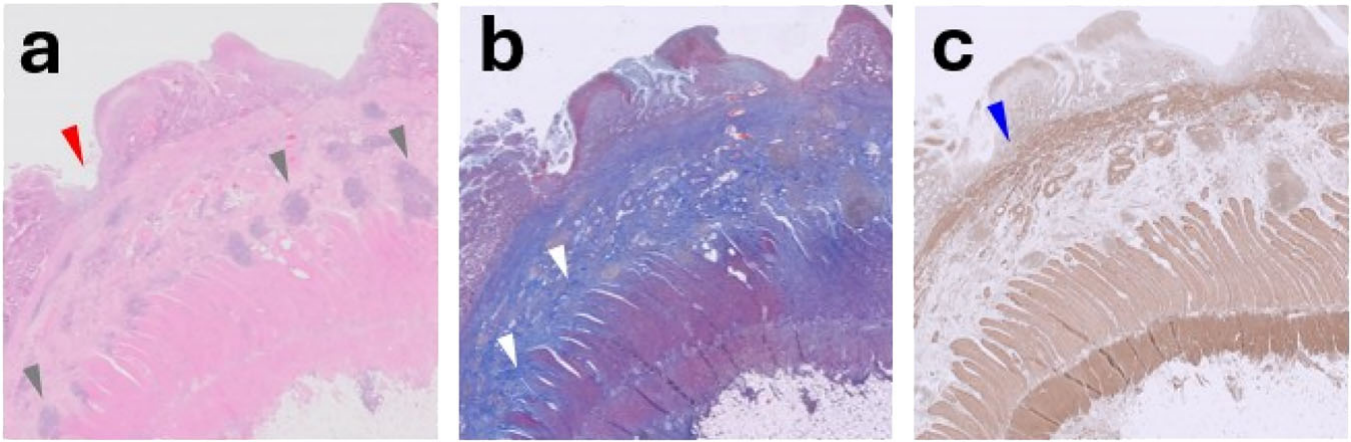
Corresponding histopathological example of a 56-year-old female with an inflammatory stricture at terminal ileum: **a** haematoxylin and eosin stain (enlargement of × 0.8) with a red arrowhead indicating a fissuring ulcer (resulting in a Nancy score of four, corresponding with severely active disease) and submucosal aggregates of lymphocytes indicated by grey arrowheads; **b** Trichrome stain with fibrotic bands crossing the midline of the submucosa (resulting in a fibrosis score of three corresponding with marked fibrosis) indicated by the white arrowheads; **c** Smooth muscle actin stain with the blue arrowhead indicating a thickened muscularis mucosae which is crossing the midline of the submucosa resulting in a score of three corresponding with marked muscularisation. No changes in the muscularis propria were visible

An example MRI series of a stricture is illustrated in Fig. 4. Figure 5 illustrated the corresponding histopathology.

**Fig. 6:**
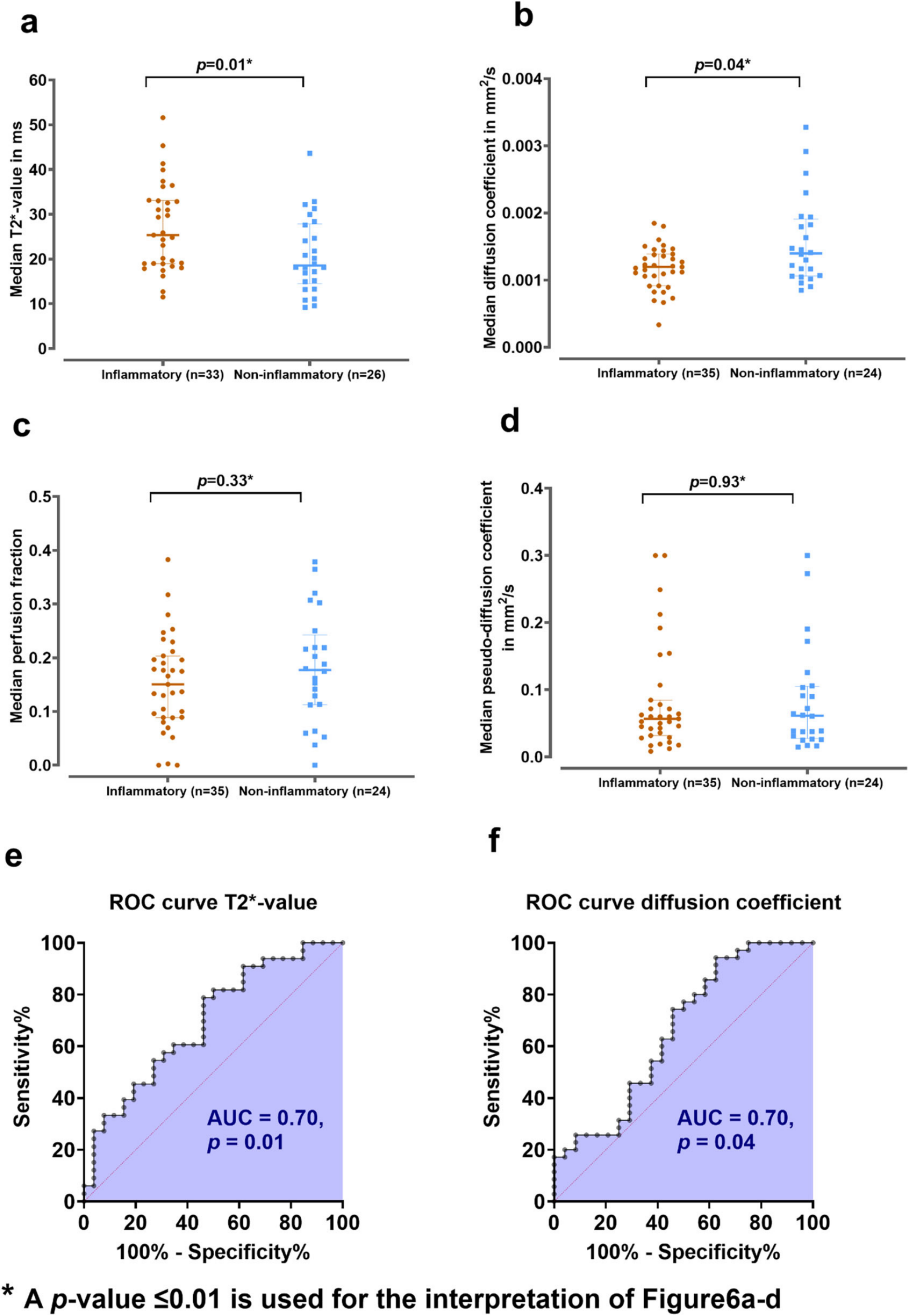
Differences between inflammatory and noninflammatory sections for different quantitative MRI parameters: **a** median T2* values in ms; **b** median diffusion coefficient in mm^2^/s; **c** median pseudodiffusion coefficient in mm^2^/s; **d** median perfusion fraction; **e** Receiver operating characteristic (ROC) analysis of T2* value; **f** ROC analysis of diffusion coefficient

Median volume of T2* segmentations was 924 mm^3^ [640‒1,253]. Median T2*-value showed a significant difference between inflammatory and noninflammatory sections (25.4 ms [19.0‒33.1], *n* = 33 *versus* 18.6 ms [14.5‒27.8], *n* = 26). Results are visualised in Fig. 6. The area under the curve (AUC) of the T2*-value for distinguishing inflammatory from noninflammatory sections was 0.70 (95% confidence interval 0.56‒0.83, *p* = 0.011), with an optimal threshold of > 18.9, showing 78.8% sensitivity and 53.8% specificity.

### Comparison of IVIM parameters with histopathological subtypes

Median volume of IVIM segmentations was 820 mm^3^ [527‒1,230]. Median diffusion coefficient was significantly lower for inflammatory sections and compared to noninflammatory sections (0.0012 mm²/s [0.0010‒0.0014], *n* = 35 *versus* 0.0014 mm²/s [0.0011‒0.0019], *n* = 24). The AUC of the diffusion coefficient for distinguishing inflammatory from noninflammatory sections was 0.70 (95% confidence interval 0.51‒0.81, *p* = 0.039), with an optimal threshold of < 0.001380, yielding a sensitivity of 74.3% and specificity of 54.2%. Median pseudodiffusion coefficient was 0.06 mm²/s [0.03‒0.08] for inflammatory sections and 0.06 mm²/s [0.03‒0.11] for noninflammatory sections (*p* = 0.926). Median perfusion fraction was 0.15 [0.09‒0.20] for inflammatory sections and 0.18 [0.11‒0.24] for noninflammatory sections (*p* = 0.327). AUC for median pseudodiffusion and perfusion fraction were 0.51 (95% confidence interval 0.35‒0.66, *p* = 0.926) and 0.58 (95% confidence interval 0.42‒0.73, *p* = 0.331), respectively.

### Comparison of conventional MRI parameters with histopathological subtypes

These results are presented in the Supplementary Results and in Table S2.

### Interobserver agreement for conventional MRI parameters and histopathology

These results are presented in the Supplementary Results and in Table S3.

## Discussion

In patients with stricturing CD, we found a significant higher T2*-value and a trend towards a lower diffusion coefficient measured with IVIM in inflammatory compared to noninflammatory sections. In contrast, the pseudodiffusion coefficient and perfusion fraction showed no difference.

It is hard to compare our results to the literature. At first, previous literature focused on fibrosis degrees and their correlation with quantitative MRI parameters [15, 16], while we identified inflammatory and noninflammatory sections of strictures histopathologically. Interestingly, we found no difference in the degree of fibrosis between inflammatory and noninflammatory sections. Second, our ROI placement differed from previous studies, as we segmented the entire bowel wall, while others used smaller, biased ROIs due to the heterogeneous composition of the bowel wall in stricturing CD [15, 16].

The higher T2*-value in inflammatory sections can be explained by more presence of oedema: 1. which was significantly more present in inflammatory compared to noninflammatory sections, 2. which showed a positive correlation with the median T2*-value in our dataset (Fig. S2). As the first to use the histopathological division between inflammatory and noninflammatory sections, our results need validation in studies using the same reference.

Analysis of T2*-maps is challenging as the T2*-value can be influenced by multiple factors, including T2 relaxation time (presence of oedema), magnetic field inhomogeneities (*e.g*., presence of deoxyhaemoglobin, presence of metal artefacts and gas-tissue transition zones), and partial volume effect of bowel lumen and bowel wall. In our dataset, we visually assessed for confounding factors (*i.e*., presence of metal artefacts, partial voluming and gas-tissue transition zones) and tried to minimise their contributions. Our results indicate that this approach enhances sensitivity to oedema, enabling distinction between inflammatory and noninflammatory sections. However, it is labour-intensive and time-consuming, which limits its application in clinical practice.

Diffusion measured with IVIM showed a trend towards a lower diffusion coefficient in inflammatory as compared to noninflammatory sections. The lower diffusion coefficient of inflammatory sections could be attributed to the significantly higher degree of inflammation and the higher muscularisation of the muscularis mucosae relative to noninflammatory sections in our series. The degree of fibrosis and muscularisation of the muscularis propria was comparable in both groups. Previous studies have shown that inflammation, fibrosis and muscularisation impair Brownian motion, resulting in reduced diffusion [17, 25, 26]. It is interesting that muscularisation of the muscularis mucosae is lower in the noninflammatory than in the inflammatory group, as this is usually seen as characteristic of chronic disease. This can be explained by our histopathological scoring system. In the inflammatory group, inflammation contributed more to the bowel wall thickness, but there were also high scores for fibrosis and muscularisation. The individual use of diffusion coefficient may be limited in clinical practice due to histopathological heterogeneity with similar findings for inflammation, fibrosis and muscularisation at diffusion.

Pseudodiffusion and perfusion fractions did not differ significantly between inflammatory and noninflammatory sections. These parameters are known to be less accurate than the diffusion coefficient, with pseudodiffusion in particular being notoriously poor [23]. Comparison with existing literature is challenging, as no studies have incorporated inflammation, fibrosis and muscularisation as histopathological reference.

Both T2*-value and diffusion coefficient exhibited a reasonable area under the curve and sensitivity, but lower specificity. This suggests limited discriminatory ability at the individual level, as evidenced by the overlap in T2*-value and diffusion coefficients between inflammatory and noninflammatory sections. During histopathological scoring, the relative contribution to bowel wall thickness of chronic features and inflammation determined the predominant stricture phenotype. We determined that muscularisation contributed more to the increase in bowel wall thickness than fibrosis in our dataset, a finding that is corroborated by earlier research [5, 6] (Fig. S1). This underlines the importance of analysing muscularisation alongside fibrosis, as muscularisation may prove to be a novel target for research in imaging biomarkers in CD.

A strength of our study is the inclusion of a histopathological reference, scored by two gastrointestinal pathologists with expertise in inflammatory bowel disease. Additionally, the matching of histopathology and MRI was carefully conducted, using the ileocecal valve or anastomosis as a reference. Another strength is the use of motion correction for DWI-IVIM. Another strength is that a second observer supervised the ROIs for correctness. However, we did not test for inter- and intraobserver reproducibility of segmentations. A further strength of our study is the imaging analysis of the entire bowel wall at the site of maximal stricturing, rather than relying on smaller ROIs. This approach avoids the potential bias introduced by the heterogeneous composition of the bowel wall in stricturing CD. Finally, a lot of effort and care went into the contouring of the strictures, ensuring only the narrowest stricture sections were included and (motion) artefacts were excluded.

Our study also has some limitations. First, there is no validated histopathological scoring system for Crohn strictures and, therefore, we deferred to a semiquantitative score. On top of that, a Nancy Index of four, indicating severe active mucosal inflammation, was scored for most strictures. This index, designed for measuring mucosal inflammation in ulcerative colitis, is not ideal for stricturing CD. Unfortunately, no more appropriate histopathology scoring method was available. As a result, a stricture with a small mucosal erosion is scored the same as one with widespread mucosal ulceration and full epithelial loss.

Second, delayed gain of enhancement [12] was not included, as it would have extended the scanning protocol duration beyond what is sustainable for patients. Third, we only included patients who were scheduled for small bowel segment resection, as histopathology was the reference. This skewed the patient population to mixed or chronic sections and resulted in the inclusion of fewer inflammatory sections. However, this is inherent to a population where histopathology is the reference. Since some patients with suspected inflammatory bowel segments were also offered to undergo surgery as first treatment and were treated with surgery [27], we were able to include some patients with solely inflammatory sections. However, this selection bias impacted our statistical power to compare solely inflammatory sections with solely chronic sections. To overcome this issue, we stratified solely inflammatory and mixed sections to the inflammatory sections and compared those with the noninflammatory sections (predominantly chronic sections). In addition, although matching between MRI and surgical resection specimen was performed as accurately as possible, it should be noted that exact matching is impossible. Due to COVID, we were not able to maintain the interval of 12 weeks between MRI and surgery; this resulted in three patients being included with a prolonged interval between MRI and surgery. Lastly, we observed that sections in stricturing CD are very heterogeneous, both within and between patients. This heterogeneity complicates the determination of a predominant histopathological phenotype and comparison with cross-sectional imaging.

In conclusion, the T2*-value was significantly higher in inflammatory sections, while the diffusion coefficient measured by IVIM showed a trend towards a lower diffusion coefficient as compared to noninflammatory sections. These parameters may potentially serve as imaging biomarkers for the distinction between inflammatory and noninflammatory strictures, pending external validation against histopathology as the reference. Given the significant overlap in histopathological features, quantifying the extent of inflammation, fibrosis and muscular hypertrophy—rather than relying on binary classification—could provide more accurate insights. A multiparametric MRI biomarker set that estimates the ratio of these features could be helpful to achieve this quantification, potentially advancing the development of MRI biomarkers for stricturing Crohn’s disease.

## Supplementary information

**Additional file 1: Table S1.** Definitions of conventional MRI parameters. **Table S2.** Differences in conventional MRI parameters between inflammatory (inflammatory and mixed) and non-inflammatory sections. **Table S3.** Interobserver agreement conventional MRI parameters and histopathological features. **Fig. S1.** Visualisation of individual scores (i.e., fibrosis and muscularisation scores) per section categorized in inflammatory versus noninflammatory sections. Per histopathological category a zero stand for no increase, one for mild increase, two for moderate increase and three for marked increase. **Fig. S2.** Correlation plot between median T2* values in ms and presence of intramural oedema measured on magnetic resonance imaging (defined as an increased signal intensity on T2-weighted image): On the x-axis, 1 = normal, 2 = minor increase in signal intensity, 3 = moderate increase in signal intensity, 4 = marked increase in signal intensity.

## Data Availability

The datasets used and analysed during the current study are available from the corresponding author on reasonable request.

## Abbreviations

AUC: Area under the curve
CD: Crohn’s disease
*D*: Diffusion coefficient
*D**: Pseudodiffusion coefficient
*f*: Pseudodiffusion signal fraction
IVIM: Intravoxel incoherent motion
MRI: Magnetic resonance imaging
ROC: Receiver operating characteristic
ROI: Region-of-interest
