# Supplementary material for: Utility of T2* and DWI-IVIM to distinguish inflammatory and noninflammatory strictures in Crohn’s disease: a prospective cross-sectional cohort study
Source: Eur Radiol Exp. 2026 Apr 22;10:52. doi: 10.1186/s41747-026-00715-0 (PMC13103191; doi:10.1186/s41747-026-00715-0)
Supplement: Supplementary file 1 — Additional file 1: Table S1. Definitions of conventional MRI parameters. Table S2. Differences in conventional MRI parameters between inflammatory (inflammatory and mixed) and non-inflammatory sections. Table S3. Interobserver agreement conventional MRI parameters and histopathological features. Fig. S1. Visualisation of individual scores (i.e., fibrosis and muscularisation scores) per section categorized in inflammatory versus noninflammatory sections. Per histopathological category a zero stand for no increase, one for mild increase, two for moderate increase and three for marked increase. Fig. S2. Correlation plot between median T2* values in ms and presence of intramural oedema measured on magnetic resonance imaging (defined as an increased signal intensity on T2-weighted image): On the x-axis, 1 = normal, 2 = minor increase in signal intensity, 3 = moderate increase in signal intensity, 4 = marked increase in signal intensity. [file 41747_2026_715_MOESM1_ESM.pdf]

Utility of T2* and DWI-IVIM to distinguish inflammatory and noninflammatory strictures in Crohn disease: a prospective cross-sectional cohort study

ELECTRONIC SUPPLEMENTARY MATERIAL

Supplementary material and methods

Collection of conventional MRI data

Two abdominal radiologists [JT and KH with 14 and 21 years of experience with MRI in CD, respectively] who were blinded for all other patient data, but aware of the study aim(s), assessed conventional MRI parameters (s*upplementary table 1*).

Supplementary results

Comparison of conventional MRI parameters with histopathological subtypes

Inflammatory sections had a significant thicker bowel wall compared to non-inflammatory sections (7.8 mm (±2.0) vs. 6.4 mm (±1.4)). A significantly increased signal intensity of the bowel wall on T2-weighted images was more present in inflammatory (n=38, 100%) compared to non-inflammatory sections (n=38, 85%). Mesenteric inflammation was significantly more present in inflammatory (n=23, 61%) compared to non-inflammatory sections (n=10, 30%) Other conventional MRI parameters showed no significant differences between inflammatory and non-inflammatory sections *(supplementary table 2).*

Interobserver agreement conventional MRI parameters

Interobserver agreement of conventional MRI parameters (*supplementary table 3*) varied between fair and substantial according to the classification of Landis and Koch (28).

Interobserver agreement histopathology

Interobserver agreement (*supplementary table 3*) for histopathological subtype and Nancy index were moderate and substantial according to the interpretation of Landis and Koch (28).

Supplementary tables

***Table S1.** Definitions of conventional MRI parameters*

MRI feature	Categories	Definition
**Bowel wall thickness**	Measured in mm	>3 mm (29)<br>Measured at the thickest part of the bowel wall. For the measurement, an axial T1-weighted post-contrast sequence was used, when unavailable or of poor quality an axial T2-weighted image with fat suppression was used.
**Intramural oedema**	- Equivalent to normal<br>- Minor increase in signal intensity<br>- Moderate increase in signal intensity<br>- Marked increase in signal intensity	Equivalent to normal bowel wall or a bowel wall that appears to be either dark grey (minor), light grey (moderate) or contains areas of white high signal approaching the appearance of luminal content (marked) on a T2-weighted image with fat suppression (30)
**Mesenteric inflammation**	- Normal<br>- Increase in mesenteric signal intensity but no fluid<br>- Small fluid rim<br>- Large fluid rim (30)	The presence of an increase in mesenteric signal and/or fluid rim assessed on a T2-weighted image with fat suppression (30).
**Presence of fat in bowel wall**	- Present<br>- Absent	The combination of the presence of hyperintensity on a regular (not fat suppressed) T2-weighted image and the absence of hyperintensity on a fat suppressed T2-weighted or enhancement on T1-weighted post-contrast image.
**T1 enhancement**	- Asymmetric<br>- Homogeneous<br>- Stratified: bilaminar or trilaminar	More enhancement at the mesenteric border compared to the anti-mesenteric border (asymmetric).<br>Homogeneous enhancement is homogeneous enhancement of all bowel wall layers (29, 31).<br>Stratified is a layered enhancement pattern that is either bilaminar (mucosal enhancement) or trilaminar (mucosal and serosal enhancement).
**T1 hyperintensity**	Equivalent to normal<br>Minor enhancement<br>Moderate enhancement<br>Marked enhancement	Equivalent to normal bowel wall, or a bowel wall with enhancement greater than normal small bowel but significantly less than nearby vascular structures (minor), or somewhat less than nearby vascular structures (moderate), or the signal approaches that of nearby vascular structures (marked) (30).

**Presence of ulcerations**	Present<br>Absent	The presence of small focal breaks in the intraluminal surface with an extension of air or enteric contrast into the affected bowel wall or lineair hyperenhancement perpendicular to the bowel wall (29)
**Presence of fatty wrapping**	Present<br>Absent	Scored as present when more than 50% of the bowel wall was surrounded by mesenteric fat (32)

Table S2. *Differences in conventional MRI parameters between inflammatory (inflammatory and mixed) and non-inflammatory sections*

**MRI feature**<br>**in mean (±SD) or n (%)**	**Inflammatory (n=38)**	**Non-inflammatory (n=33)**	***p*-value**
**Bowel wall thickness**	7.8 (±2.0)	6.4 (±1.4)	0.001
**Intramural oedema**			0.018
Equivalent to normal	0 (0)	5 (15)	
Increased signal intensity of the bowel wall on T2-weighted MRI	38 (100)	28 (85)	
Minor increase in signal intensity	6 (16)	10 (30)	
Moderate increase in signal intensity	28 (74)	18 (55)	
Marked increase in signal intensity	4 (10)	0	
**Mesenteric inflammation**			0.017
Normal	15 (39)	23 (70)	
Mesenteric inflammation	23 (61)	10 (30)	
Increase in mesenteric signal but no fluid	14 (36)	5 (15)	
Small fluid rim	3 (8)	3 (9)	
Large fluid rim	6 (16)	2 (6)	
**Presence of fat in bowel wall**	6 (16)	7 (21)	0.56
**T1 enhancement**			0.82
Homogeneous	2 (5)	2 (6)	
Stratified: bilaminar	6 (16)	7 (21)	
Stratified: trilaminar	30 (79)	24 (73)	
**T1 hyperintensity**			0.83
Minor enhancement	0	2 (6)	
Moderate enhancement	19 (50)	14 (42)	
Marked enhancement	19 (50)	17 (52)	
**Presence of ulcerations**	34 (90)	25 (76)	0.12
**Presence of fatty wrapping**	34 (90)	27 (82)	0.36

Table S3. *Interobserver agreement conventional MRI parameters and histopathological features*

**Conventional MRI/histopathological feature**	**Interobserver agreement [illegible] or ICC (95% CI)**	**Interpretation***
Bowel wall thickness	0.75 [0.59-0.85]	Substantial
Intramural oedema	0.41 [0.21-0.60]	Moderate
Mesenteric inflammation	0.71 [0.54-0.88]	Substantial
Fat in wall	0.37 [0.12-0.61]	Fair
T1 enhancement	0.30 [0.04-0.56]	Fair
T1 hyperintensity	0.49 [0.34-0.63]	Moderate
Ulcerations	0.33 [0.10-0.55]	Fair
Fatty wrapping	0.57 [0.35-0.79]	Moderate
Histopathological subtype	0.45 [0.25-0.66]	Moderate
Nancy index	0.78 [0.65-0.91]	Substantial

* According to Cohen’s k interpretation guideline Landis and Koch (28)

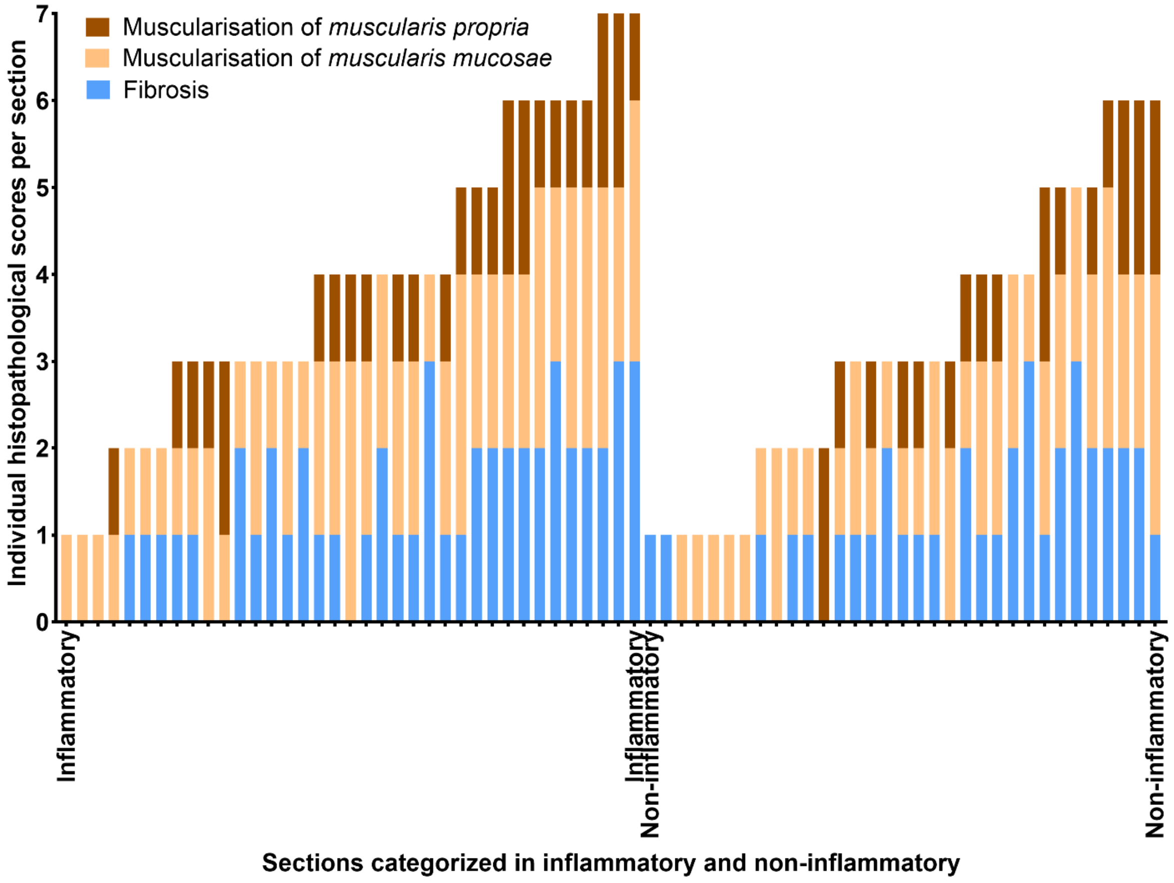

Fig. S1 Visualization of individual scores (*i.e.*, fibrosis and muscularisation scores) per section categorized in inflammatory *versus* noninflammatory sections. Per histopathological category a zero stand for no increase, one for mild increase, two for moderate increase and three for marked increase

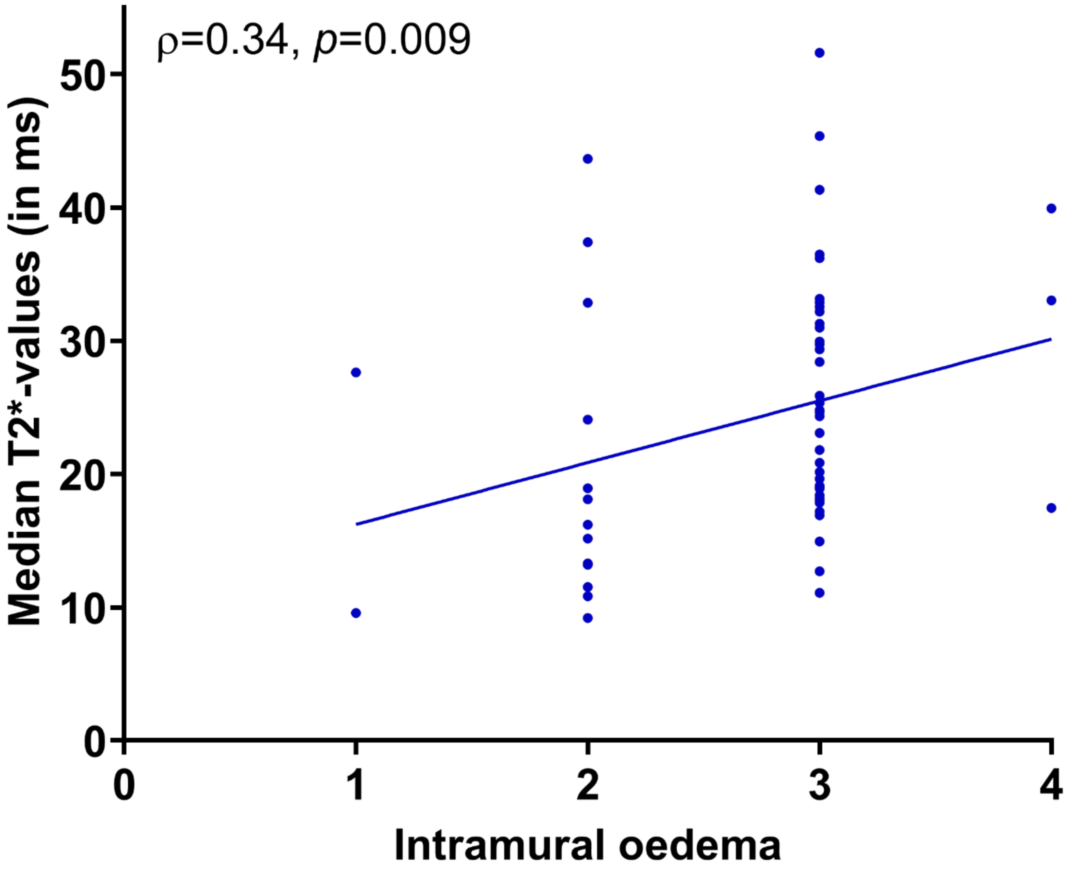

Fig. S2. Correlation plot between median T2* values in ms and presence of intramural oedema measured on magnetic resonance imaging (defined as an increased signal intensity on T2-weighted image): On the *x*-axis, 1 = normal, 2 = minor increase in signal intensity, 3 = moderate increase in signal intensity, 4 = marked increase in signal intensity.
